# Intermediate-Risk Pulmonary Embolism: A Review of Contemporary Diagnosis, Risk Stratification and Management

**DOI:** 10.3390/medicina58091186

**Published:** 2022-08-30

**Authors:** Akshay Machanahalli Balakrishna, Vuha Reddi, Peter Matthew Belford, Manrique Alvarez, Wissam A. Jaber, David X. Zhao, Saraschandra Vallabhajosyula

**Affiliations:** 1 Department of Medicine, Creighton University School of Medicine, Omaha, NE 68124, USA; 2 Department of Medicine, Danbury Hospital/Yale University School of Medicine, Danbury, CT 06810, USA; 3 Section of Cardiovascular Medicine, Department of Medicine, Wake Forest University School of Medicine, Winston-Salem, NC 27262, USA; 4 Section of Cardiovascular Medicine, Department of Medicine, Emory University School of Medicine, Atlanta, GA 30307, USA; 5 Department of Implementation Science, Division of Public Health Sciences, Wake Forest University School of Medicine, Winston-Salem, NC 27262, USA

**Keywords:** pulmonary embolism, sub-massive, intermediate risk, biomarkers, risk stratification, management

## Abstract

Pulmonary embolism (PE) can have a wide range of hemodynamic effects, from asymptomatic to a life-threatening medical emergency. Pulmonary embolism (PE) is associated with high mortality and requires careful risk stratification for individualized management. PE is divided into three risk categories: low risk, intermediate-risk, and high risk. In terms of initial therapeutic choice and long-term management, intermediate-risk (or submassive) PE remains the most challenging subtype. The definitions, classifications, risk stratification, and management options of intermediate-risk PE are discussed in this review.

## 1. Introduction

Pulmonary embolism (PE) is the third most common cause of death among hospitalized patients [1]. PE can be classified into low-risk, intermediate-risk (or submassive), and high-risk PE based on the hemodynamic stability and presence of right ventricular strain. Patients are considered hemodynamically unstable if systolic blood pressure (SBP) is <90 mmHg or a drop in SBP of ≥40 mm Hg or more from baseline or hypotension requires inotropes or vasopressors. Among hemodynamically stable patients, PE is described as low-risk if there is no evidence of right heart strain, and intermediate-risk in the presence of right heart strain. PE is stratified as high-risk when there is hemodynamic instability. Though often the management of high- and low-risk PE is reasonably well understood, our understanding on the management and best practices in intermediate-risk remains limited. Mortality from intermediate-risk PE has been reported to range from 3 to 14% [2,3,4,5,6]. Efforts to improve prognosis in people with intermediate-risk PE have yielded limited success due to the wide variability in the presentation and diagnosis of these patients [7]. Limited and underpowered research studies, different definitions of intermediate-risk PE, and non-standardized right ventricular dysfunction (RVD) parameters are some of the main challenges faced. Furthermore, there is limited information about predictors of decompensation versus stability in this population which makes risk prediction challenging in the short and intermediate term [8]. We seek to explain the present evidence and inform clinical practice with this publication. To explore treatment recommendations, we review the definitions, prognostic variables, risk stratification, and management of intermediate-risk PE.

## 2. Definitions and Classification

The term “intermediate-risk PE” refers to a population of patients whose illness severity falls between that of massive PE, which is marked by hemodynamic instability, and standard low-risk PE. Because of its crucial role in disease severity, RVD is vital to this risk-stratification. In defining intermediate-risk PE, RVD is frequently paired with elevated cardiac biomarkers. However, whether one or both conditions are essential has been subject to significant discussion in the literature. The American Heart Association (AHA), American College of Chest Physicians (ACCP), and European Society of Cardiology (ESC) have all issued varying guidelines that emphasize this heterogeneity (Table 1).

Intermediate-risk PE is defined in the AHA guidelines as acute PE without systemic hypotension but with evidence of RVD, which can be identified by echocardiography or computed tomography (CT) imaging, biomarkers indicative of right ventricular (RV) strain, and/or ischemia (brain natriuretic peptide (BNP) or troponin), and/or particular changes seen on electrocardiography (T wave inversions in the right precordial leads V1–V4 ± the inferior leads II, III, and aVF) [3]. The ACCP definition of intermediate-risk PE is fulfilled if either RVD or abnormal cardiac biomarkers are present (including abnormal BNP in addition to elevated troponins) [9]. The ESC guidelines [10] divide intermediate-risk PE into two categories: intermediate-high risk and intermediate-low risk. The ESC incorporates scoring systems such as Pulmonary Embolism Severity Index (PESI) and simplified PESI (sPESI) in defining intermediate-risk PE. Low risk is defined by PESI class III/IV or simplified PESI score ≥ 1 and with or without one of either (1) Evidence of RVD on imaging (CT/Echocardiography) or (2) an elevated RV biomarker (troponin or BNP). High risk is defined by PESI class III/IV or sPESI score ≥1, in addition to imaging evidence of RVD (as seen on CT or echocardiography), and an elevated RV biomarker (troponin or BNP).

## 3. Approach to Risk Stratification

**Figure 1:**
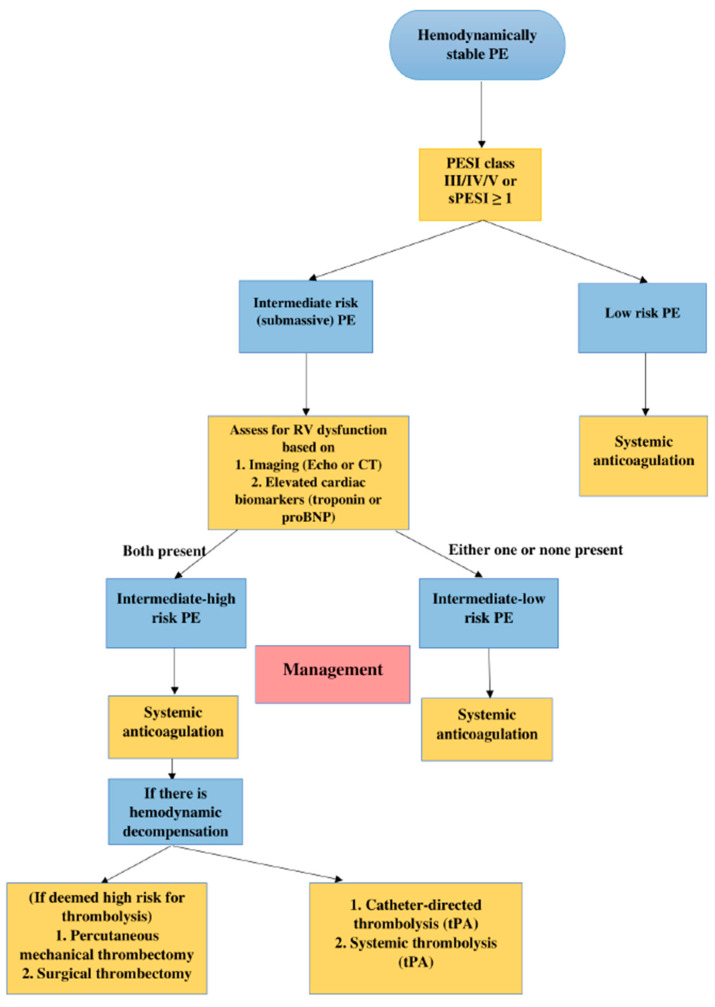
Approach to risk stratification and risk-based management of hemodynamically stable acute PE syndromes. BNP: brain natriuretic peptide; CT: computed tomogram; PE: pulmonary embolism; PESI: Pulmonary Embolism Severity Index; RV: right ventricular; sPESI: simplified Pulmonary Embolism Severity Index; tPA: tissue plasminogen activator.

For individuals with intermediate-risk PE, current ESC guidelines recommend a progressive combination of clinical scoring systems, biomarkers, and imaging (Figure 1).

Intermediate risk PE encompasses a diverse population and a wide spectrum of severity, from patients who can be managed with a short hospital stay to those who need close monitoring for potential rescue thrombolysis, aspiration thrombectomy, catheter-directed thrombolytics, or surgical embolectomy. Nevertheless, the management of this population can be most challenging because a subset may suddenly develop profound arterial hypotension, obstructive or cardiogenic shock, and sudden death without ominous signs despite prompt therapeutic anticoagulation [11]. Therefore, it is essential to use appropriate risk stratification tools to guide treatment decisions.

## 4. Risk Stratification Tools

### 4.1. Right Ventricular Dysfunction

The abrupt increase in pulmonary artery pressure from PE results in an increased RV afterload along with consequent elevation of right ventricular tension. As the afterload increases, the right ventricle dilates with resultant left ventricular (LV) underfilling and reduction in coronary artery blood supply. These changes result in decreased RV perfusion and subsequent dysfunction from ischemia. RV dysfunction identified by computed tomography angiogram (CTA) or echocardiogram is associated with adverse clinical outcomes [12,13,14,15]. CTA and echocardiogram are thus used in risk stratifying PE based on the presence of RV dysfunction [10]. RV enlargement, defined as an RV diameter to LV diameter ratio of >0.90 based on measurements from a CTA or echocardiogram is indicative of RV dysfunction. CTA is more widely accessible than echocardiography and is often the first imaging modality pursued in this clinical scenario. The most predictive indicator is the RV/LV ratio [13,16,17,18] as determined on transverse sections, and an RV/LV ratio ≥ 0.9 was linked to an elevated risk of clinical deterioration and mortality in prior studies [18]. The predictive value of several CTA factors was assessed by a meta-analysis [13], and the following factors were known to be associated with increased all-cause mortality: An RV/LV ratio of ≥1, bowing of the interventricular septum, and contrast reflux into the inferior vena cava. These parameters’ predictive value only applies to patients with unselected PE; hence, it might not be able to accurately prognosticate patients with very low-risk condition. This is confirmed by the findings of a prior study where an RV/LV of ≥0.9 or ≥1.0 was not linked to worse outcomes in patients with a sPESI score of zero [17]. Additional echocardiographic findings indicative of RVD include flattening of the interventricular septum and contrast reflux into the inferior vena cava and hepatic veins and McConnell’s sign (decreased RV free wall function with apical sparing) [19]. RV myocardial function (Tei index), RV longitudinal strain, and fractional area change may be useful in risk stratification; however, they are time-consuming and may be difficult to obtain in acutely ill patients.

### 4.2. Biomarkers

Several biomarkers have been suggested for diagnosis and risk stratification of PE in the modern era.

#### 4.2.1. Cardiac Troponins

Cardiac troponin T is a highly sensitive and specific marker of myocardial cell injury and is being used as the preferred biomarker for the diagnosis of myocardial infarction. Theoretically, the release of cardiac troponin from the myocardium results from acute RV pressure overload, impaired coronary blood flow, and severe hypoxemia caused by PE. Elevated troponin was associated with a significant risk of short-term mortality and adverse outcomes in a recently conducted meta-analysis [12]. Troponin testing has been shown to improve the predictive value of echocardiography in PE, and biomarkers have been successfully integrated into echocardiography-based risk assessment systems [3,10,20]. Although there is some evidence regarding the feasibility of elevated troponin in the prediction of mortality and complications in normotensive patients with PE [21] in the setting of troponin elevation out of proportion to PE, acute coronary syndrome and alternate etiologies should be investigated. Large multicenter studies’ results have repeatedly demonstrated that high-sensitivity cardiac troponin assays improve myocardial infarction diagnosis at the time of presentation compared to conventional assays, particularly in patients who present soon after the onset of chest pain [19]. They also enable a quicker “rule-in” and “rule-out” of myocardial infarction as they have a high negative predictive value at low levels (<14 ng/L) [22]. The fact that many low-risk PE patients die or develop RVD suggests a care gap, and laboratory testing, particularly high-sensitivity cardiac troponin assays, may be useful in filling this gap.

#### 4.2.2. Natriuretic Peptides

BNP and N-terminal pro-brain natriuretic peptide (NT-proBNP) are markers of ventricular dysfunction and pressure overload. Numerous studies have found a link between high BNP or NT-proBNP concentrations and clinical worsening in patients with acute PE [3,20,23,24]. Low-risk patients are identified by BNP values of 50 or 90 ng/L; high-risk patients have BNP values of >500 ng/L [3,23]. Small sample numbers and variances in patient characteristics are likely to be the cause of reported differences in cutoff values when using the same test. The use of echocardiography to identify a high-risk subpopulation in patients with high values is beneficial. RV dysfunction and a bad prognosis are indicated by persistent elevations in NT-proBNP (values > 7500 ng/L after 24 h or a 50% drop) [25]. However, increased natriuretic peptide concentrations have a low positive predictive value since they can occur in a variety of other conditions.

#### 4.2.3. Others

Several other biomarkers have been utilized recently in risk stratification and assist therapeutic decision-making. Heart-type fatty acid binding protein (h-FABP) is one of the most abundant non-enzyme proteins in the heart, and is produced in response to myocardial injury and has been shown to be sensitive and specific in sensing myocardial damage [26,27]. It has also been shown to be beneficial in predicting poor outcomes in patients with acute PE in various studies [3,10,27,28]. Dellas et al. described the utility of h-FABP in the risk stratification of acute normotensive PE and found that elevated h-FABP levels on admission were strongly associated with the 30-day mortality and adverse event rate. h-FABP appeared to have a better prognostic value than previously validated biomarkers such as troponin and pro-BNP, as well as the detection of RV dysfunction on echocardiography. Furthermore, high h-FABP levels at the time of presentation were linked to increased long-term mortality after acute PE [27]. Lauque et al. in 2015 through their prospective study showed that h-FABP was a stronger predictor of adverse outcomes compared to troponin and BNP, further strengthening the importance of h-FABP in the risk stratification of PE [28]. According to ESC guidelines circulating h-FABP levels ≥6 ng/mL had a negative predictive value of 99% in normotensive patients [10,28]. Kidney dysfunction has been seen in individuals with acute PE before, and it has been linked to poor short-term adverse outcomes. Plasma neutrophil gelatinase-associated lipocalin and cystatin C are excellent biomarkers of kidney dysfunction. In a prospective observational study of 142 patients, elevated neutrophil gelatinase-associated lipocalin and cystatin C levels were found to be significant predictors of all-cause and PE-related 30-day mortality. Plasma cystatin C was the strongest renal biomarker which independently predicted mortality on multivariable analysis [29]. These results indicate that serum neutrophil gelatinase-associated lipocalin and cystatin C may have a predictive function in acute PE [29]. growth differentiation factor 15 is a cytokine produced in the heart because of excess pressure or ischemia and has been shown to have a prognostic value in PE [30]. Lankeit et al. through a prospective study demonstrated that increased growth differentiation factor 15 was found to be an independent predictor of adverse 30-day outcomes in a prospective investigation of 123 individuals. Growth differentiation factor 15 also intensified the adverse outcome predictive ability of cardiac troponin, NT-proBNP, and echocardiographic findings of RVD [30].

### 4.3. Bedside Scoring Systems

Since prior biomarker-based risk stratification algorithms failed to identify people at intermediate risk who might benefit from thrombolytics [31], new clinical risk scores for predicting 30-day mortality were created and validated [32,33,34,35,36]. These scoring tools add value because they allow for the identification of patients at low risk who can be discharged and treated outpatient, as well as patients at intermediate-to-high risk who need to be monitored for hemodynamic decompensation and the need for rescue reperfusion therapy [32,33,34,35,36].

PESI and sPESI are proven risk stratification tools for patients with PE based on clinical indicators and are used at the bedside for prognostication of the risk of adverse events and therapeutic decision making. Patients with high PESI and sPESI scores have a higher 30-day mortality rate. These measures, in combination with an evaluation of RV function, have been used to classify patients as intermediate-low risk or intermediate-high risk, and to aid in treatment decisions [34,37,38]. Further risk assessment must involve imaging evidence of RV dysfunction by echocardiography or CTA within the intermediate-risk group (PESI III or sPESI 1). The 2019 ESC guidelines for the management of acute PE included the PESI or sPESI [10]. Biomarker testing thus adds to the prognostic knowledge. As a result, patients with elevated cardiac biomarker concentrations, especially a positive cardiac troponin test, and imaging evidence of RVD should be classed as intermediate-high risk. These patients must be closely monitored for the first two to three days to detect hemodynamic decompensation and the necessity for rescue reperfusion therapy [10].

The Bova score was created to assess which patients with PE who were hemodynamically stable had inferior outcomes. It is a seven-point scoring system consisting of four clinical variables: heart rate ≥ 110 beats per minute (1 point), SBP of 90–100 mmHg for at least 15 min (2 points), RV dysfunction (2 points), and high cardiac troponin (2 points) [36,39]. This scoring system divides normotensive PE patients into three groups: stage I (0–2 points), stage II (3–4 points), and stage III (>4 points), with 30-day composite PE-related adverse complication rates of 4.2 percent, 10.8 percent, and 29.2 percent, respectively, while 30-day PE related mortality was 1.7%, 5.0% and 15.5% for stages I, II and III, respectively [36]. The score can also be interpreted as low risk (0–2), intermediate risk (2–4), and high risk (>4). A meta-analysis of nine studies confirmed the effectiveness of the Bova score in differentiating normotensive PE with different short-term prognosis and recognizing patients at higher risk of short-term adverse events [40].

## 5. Management

Intermediate risk PE is managed mainly by systemic anticoagulation. Other options include catheter-based therapy, systemic thrombolysis, and surgical embolectomy. Risk-based management of intermediate-risk PE is described in Figure 1. A synopsis of advantages and disadvantages associated with major therapeutic strategies utilized in intermediate-risk PE is described in Table 2.

### 5.1. Systemic Anticoagulation

Anticoagulation remains the cornerstone of treatment for patients with intermediate-risk PE. There are no clear recommendations on the anticoagulant of choice. The initial choice of anticoagulation in patients with intermediate-risk PE is generally between low-molecular weight heparin and a direct oral anticoagulant, as these are associated with a lower risk of bleeding compared to unfractionated heparin [41,42]. However, a patient on the verge of hemodynamic compromise may be best served by intravenous unfractionated heparin as they might require additional therapy with fibrinolysis, catheter-based intervention, or surgery.

If intermediate high-risk PE patient has any of the following characteristics of decompensation including moderate to severe RV systolic dysfunction, moderate to severe tachycardia, tachypnea, or hypoxemia, borderline hypotension, extensive embolic burden/residual deep vein thrombosis (DVT), cardiovascular comorbidities, it warrants escalation of care. Those patients without deterioration can be switched to oral anticoagulation after observation for 24 to 48 h [43].

At the time of clinical deterioration of intermediate high-risk PE patients, initiation of rescue reperfusion therapy is recommended. This may be in the form of systemic thrombolysis, catheter-directed thrombolysis, or surgical embolectomy [10].

### 5.2. Systemic Thrombolysis

Rapid thrombus clearance is achieved using systemic thrombolysis, which improves pulmonary pressure, RVD, and hemodynamics more quickly than anticoagulation alone [41,42]. While patients with major PE have a lower mortality rate, the benefit in patients with intermediate-risk PE is unclear [31,44,45,46]. This is because randomized studies that compared heparin with alteplase in intermediate-risk PE patients showed there was reduced escalation to emergency treatment without affecting mortality in patients receiving thrombolytic treatment [45]. Therefore, the guidelines do not recommend the routine use of systemic thrombolysis in intermediate-risk PE [9]. 

The Pulmonary Embolism Thrombolysis (PEITHO) trial [31], the largest randomized controlled trial of systemic thrombolysis in PE to date, compared the effects of systemic thrombolysis with tenecteplase followed by anticoagulation with heparin versus heparin alone on the primary outcome of all-cause mortality or hemodynamic collapse within 7 days of randomization. The study concluded that although hemodynamic decompensation was significantly less in the thrombolysis group, higher rates of major bleeding (defined as per the criteria of the International Society on Thrombosis and Hemostasis [47] as fatal bleeding and/or symptomatic bleeding in a critical area or organ, and/or bleeding causing a fall in hemoglobin level of 20 g/L or more or leading to transfusion of two or more units of whole blood or red cells) was observed. No reduction in mortality at day 7 or day 30 was noted in the thrombolysis arm [31]. Three years follow-up showed no difference in mortality or incidence of chronic thromboembolic pulmonary hypertension between the two groups [48]. The North American Tenecteplase or Placebo: Cardiopulmonary Outcomes at Three Months (TOPCOAT) trial also investigated thrombolysis in patients with intermediate-risk PE and compared low molecular weight heparin and tenecteplase with low molecular weight heparin [46]. Better functional capacity and quality of life at 3 months with fewer adverse outcomes were noted in the systemic thrombolytic group. However, this study could not demonstrate benefit in mortality due to low power and early termination of the trial [46].

In all the studies and meta-analyses conducted to date on systemic thrombolysis, mortality benefits in intermediate-risk PE remain debatable. However, most of the studies showed a significant association with increased bleeding including intracranial hemorrhage. This was especially noted to be true with increasing age [49]. Thrombolysis does have a short-term benefit and can be considered in patients under 75 years of age. To offset the risk of major bleeding with systemic thrombolysis, some potential solutions include reduced-dose thrombolysis and catheter-based interventions.

### 5.3. Reduced Dose Thrombolytics

The MOPETT (Moderate pulmonary embolism treated with thrombolysis) trial evaluated the role of lower dose thrombolysis along with anticoagulation vs. anticoagulation alone. A reduction in pulmonary artery hypertension was noted which was immediate and lasted the duration of the study of 28 months. The study concluded that the lower dose is safe and effective in the management of moderate PE [50]. Another study compared low dose thrombolysis plus low molecular weight heparin (LMWH) to LMWH alone in intermediate-risk PE. 38 patients were enrolled in each of the two groups. The study group had significantly fewer deaths and hemodynamic compromises at 30 days without increasing the risk of bleeding [51].

There has also been mention of using a quarter of the standard thrombolysis dose with a successful reduction in pulmonary artery pressure and right ventricle/left ventricle ratio. The treatment in this study included 10 mg of tissue plasminogen activator (tPA) given in 1 min followed by 15 mg in 2 h. Unfractionated heparin was given for 24 h followed by a direct oral anticoagulant which was used for maintenance therapy thereafter [49].

In order to test the hypothesis of similar efficacy and reduced bleeding risk with half-dose thrombolysis, Kiser et al. compared half-dose fibrinolysis versus full-dose fibrinolysis in acute PE patients. The study included 3768 patients, out of which about 700 patients received half-dose alteplase while the rest received full-dose alteplase. Half-dose fibrinolysis group showed an increased frequency of treatment escalation requiring secondary fibrinolysis and catheter directed treatment. Hospital mortality, intracranial hemorrhage, GI bleeding and blood loss anemia were similar between the two groups [52]. Thus, the safety and efficacy of half-dose thrombolysis remains questionable, needing further studies prior to recommending its use in the management of PE.

### 5.4. Catheter-Directed Therapy

Catheter-directed therapy (CDT) includes catheter-based thrombolysis and mechanical aspiration thrombectomy. Catheter-directed thrombolysis depends on the location of the clot as the embolus must be proximal for the therapy to be effective, especially for aspiration thrombectomy. The effectiveness and safety of CDT in the management of intermediate-risk PE were demonstrated in the PERFECT (Pulmonary Embolism Response to Fragmentation, Embolectomy, and Catheter thrombolysis) trial. It studied patients with acute PE and their management with CDT. Clinical success defined as hemodynamic stability, improvement in pulmonary hypertension or right heart strain, or both were achieved with no major procedure-related complications or major bleeds [53,54].

CDT includes low-dose fibrinolytic agents injected over 12 to 24 h into the pulmonary artery directly via a standard catheter or ultrasound-assisted thrombolysis (USAT). SUNSET sPE (Standard vs. Ultrasound-Assisted Catheter thrombolysis for Submassive Pulmonary Embolism) trial randomized patients with intermediate-risk PE to standard catheter-directed thrombolysis or ultrasound-assisted thrombolysis. Both arms received a similar dose and duration of alteplase resulting in comparable pulmonary artery thrombus reduction [55]. Although ultrasound increases the efficacy of thrombolysis theoretically, USAT does not confer such an advantage over traditional CDT clinically [49,55].

CDT in the treatment of intermediate-risk PE was evaluated in the ULTIMA (ultrasound accelerated thrombolysis of pulmonary embolism) study [56]. In this study, patients were randomized to receive heparin alone or heparin with catheter-directed thrombolysis (tPA and USAT) with the primary endpoint of RV dysfunction measured by the difference in RV by LV ratio on echo between presentation to 24 h later. Secondary outcomes included 90-day mortality, recurrent venous thromboembolism, and bleeding. There was an improvement in the RV dysfunction index at 24 h in the heparin plus CDT arm; however, this did not hold true at 90 days. There was no difference in secondary outcomes between the two groups [56].

SEATTLE II (A trial of EkoSonic endovascular system and Activase for treatment of acute pulmonary embolism) trial which included predominantly intermediate-risk PE patients proved a decrease in RV dysfunction by CT scan at 48 h in the group receiving CDT/USAT with anticoagulation. Major bleeding events were noted to be more likely in high-risk PE patients [57]. OPTALYSE (optimal duration of requested pulse thrombolysis procedure and acute intermediate-risk pulmonary embolism) trial demonstrated that even a lower dose thrombolytic agent with ultrasound-assisted catheter thrombolysis for a short period of 6 to 12 h improved RV strain and decreased RV afterload [58].

The optimal patient for catheter-directed therapy based on the studies is an intermediate-risk PE patient who is on the verge of hemodynamic compromise [59]. The above trials showed acute short-term benefits in reduction in RV dysfunction; however, none of them were designed to compare long-term effects such as chronic thromboembolic pulmonary hypertension or post PE syndrome [49,60]. The main clinical outcome of interest, especially in the short term for patients with intermediate-risk PE, is mortality. Based on available data there is no mortality benefit in intermediate-risk PE patients receiving CDT [49].

Moving forward catheter mediated thrombolysis may be more frequently used. The knowledge gaps in areas of time to catheter placement, mortality data, cost, and clinical outcomes compared to anticoagulation challenge use in clinical practice, especially in intermediate-risk PE patients [61].

Mechanical removal of clots can be achieved with different devices. Rheolytic thrombectomy removes the thrombus by creating negative pressure force using a saline jet at high pressure. This technique sometimes releases adenosine leading to hemodynamic decompensation [62]. United States Food and Drug Administration issued a black box based on safety concerns, suggesting the AngioJet device should not be used as the initial treatment in these patients [63]. The FLARE (FlowTriever Pulmonary Embolectomy clinical study) study on mechanical thrombectomy with the FlowTriever System in intermediate-risk PE patients noted an adverse event rate of 3.8% while gaining significant improvement in RV/LV ratio at 48 h [64]. The more recent data on the interim results of the FlowTriever All-Comer Registry for Patient Safety and Hemodynamics (FLASH) registry proved a significant immediate hemodynamic improvement while on-table for the procedure and reduced admitted days in the hospital. The results also emphasize the safety of the FlowTriever system with 0.2% all-cause mortality at the 6-month mark. Based on these results, PEERLESS, a randomized controlled trial comparing FlowTriever and catheter-directed thrombolysis was announced and is underway [65].

### 5.5. Thrombolysis versus Systemic Anticoagulation

The benefits of thrombolysis (systemic or catheter-directed) compared to anticoagulation alone, in intermediate-risk PE remains controversial. While patients with massive PE have improved mortality rates [66,67,68], its benefit in patients with intermediate-risk PE has not been clearly demonstrated [31,45,46,69]. An overview of the major trials comparing these two strategies is described in Table 3.

A large number of systematic reviews and meta-analyses have evaluated the benefits and harms of thrombolytic therapy in PE. However, meta-analyses in intermediate-risk PE have generated conflicting results. For example, a meta-analysis of 16 randomized controlled trial (RCT) studies by Chatterjee et al. in 2014 demonstrated that thrombolysis decreased all-cause mortality in intermediate-risk PE [70]. Thrombolytic therapy was associated with lower all-cause mortality (2.17% vs. 3.89%, OR 0.53, 95% CI 0.32–0.88) and an increased risk of major bleeding (OR, 2.73; 95% CI, 1.91–3.91; 9.24% vs. 3.42%). The prespecified analysis of eight studies that only included patients with intermediate-risk PE sustained the reduction in all-cause mortality (OR 0.48, 95% CI 0.25–0.92; 1.39% vs. 2.92%). However, a meta-analysis by Nakamura et al. in the same year reported different outcomes [71]. Six RCTs that compared thrombolysis with heparin in intermediate-risk PE constituted the meta-analysis. There was no significant difference in all-cause mortality (Relative risk [RR] 0.72, 95% CI 0.39–1.31; 2.3% vs. 3.7%) or major bleeding (RR 2.07, 95% CI 0.58–7.35; 6.6% vs. 1.9%) between the two groups. The authors concluded that thrombolysis did not lower the risk of death in intermediate-risk PE. The inclusion of many primary studies is one rationale for the conflicting outcomes. Eight additional RCTs that were not qualified for the Nakamura et al. review, including MOPETT and ULTIMA [50,56], were included by Chatterjee et al. in the intermediate-risk PE meta-analysis. Other than that, the primary research shared by the two metanalyses were identical. It was unique to include MOPETT since it only included individuals with “moderate” PE, which was determined by clot burden rather than RVD or positive cardiac biomarkers. Additionally, the short-term mortality was not assessed, and the deaths occurred after a 28-month follow-up. The ULTIMA study’s inclusion was particularly unusual because it focused on CDT rather than systemic thrombolysis [56].

Furthermore, the ULTIMA trial [56] remains the only randomized controlled trial to date to compare outcomes of catheter-directed thrombolysis plus anticoagulation with anticoagulation (heparin) alone. A recent meta-analysis [72] that included both observational studies and the ULTIMA trial showed that, catheter-directed thrombolysis was associated with significantly lower in-hospital mortality (RR 0.41, 95% CI 0.30 to 0.56, *p* < 0.00001), 30-day mortality (RR 0.37, 95% CI 0.18 to 0.73, *p* = 0.004), 90-day mortality (RR 0.36, 95% CI 0.17 to 0.72, *p* = 0.004), and a tendency toward lower 1-year mortality (RR 0.56, 95% CI 0.29 to 1.05, *p* = 0.07) when compared with anticoagulation alone. The risks of major bleeding (RR 1.31, 95% CI 0.57 to 3.01, *p* = 0.53), minor bleeding (RR 1.67, 95% CI 0.77 to 3.63, *p* = 0.20), and the rates of blood transfusion (RR 0.34, 95% CI 0.10 to 1.15, *p* = 0.08) were similar between the two strategies.

### 5.6. Surgical Embolectomy

Surgical embolectomy can be conducted up to the level of the segmental pulmonary arteries. It can be used when thrombolysis is contraindicated; however, its use varies widely by center and operator.

A study on the New York state registry showed lower rates of stroke, need for reintervention, and recurrent PE in patients undergoing surgical thrombectomy for treatment of acute PE. However, there is no difference in short-term mortality when compared to systemic thrombolysis [73] Surgical embolectomy has been renewed over time and the technique revised to make it a safe procedure. Data in massive PE have shown in-hospital mortality to be as low as 11.7% and another study no mortality in the surgical cohort [74,75]. Such data are lacking in intermediate-risk PE.

## 6. Additional Treatment Options

Additional treatment strategies that have been tested include mechanical support devices, inferior vena cava (IVC) filters, IV nonsteroidal anti-inflammatory drugs, and pulmonary arterial vasodilators. Some newer treatment options are also mentioned below. 

### 6.1. Mechanical Support Devices

Intermediate-risk PEs have apparent hemodynamic stability but often have hemodynamic characteristics consistent with cardiogenic shock, despite normal blood pressure and heart rates at presentation [76]. Mechanical hemodynamic support can be used in the management of PE on the cusp of cardiac arrest, and/or in the presence of contraindications to or failure of systemic thrombolysis [77].

In patients with impending decompensation, veno-arterial-extracorporeal membrane oxygenation (ECMO) alone or as part of reperfusion strategy along with other management options might offer survival benefits compared to thrombolysis alone [78]. ECMO has only been described sparingly in the PE population, especially the intermediate-risk PE population. ECMO also acts as a bridge to RV recovery while other strategies are implemented to reduce the thrombus burden. In a retrospective study conducted at a tertiary care center, four patients were included in the analysis to study the role of ECMO in PE patients. Three of the four patients had massive PE while one had intermediate-risk PE. One of them who was otherwise expected to die survived. This highlighted the need to study risk factors and outcome predictors with further studies [79].

Impella may be reasonable in scenarios where RVD persists the following reperfusion. The risk of bleeding may be lower compared to ECMO and may allow utilization with thrombolytics [76]. An Impella device has no oxygenators and therefore limits its use in severely hypoxic patients [77]. A case series on five patients with shock due to massive or intermediate-risk PE resulted in immediate hemodynamic benefit and survival to discharge with Impella RP device implantation [80].

In a case report, an intermediate-risk PE patient underwent surgical embolectomy under extracorporeal circulation owing to the presence of a right atrium thrombus. This patient developed acute right heart failure that resolved with a temporary right ventricular assist device (RVAD) support. RVAD may have utility as demonstrated in this report after reperfusion therapy for the management of RV failure [81].

Tandem heart is an extracorporeal centrifugal pump to which an oxygenator may be added if necessary [77]. Its place in PE management is not widely studied and calls for further studies to explore the role of mechanical devices in the management of intermediate-risk PE.

### 6.2. Inferior Vena Cava Filter

IVC filters can be considered if the patient has recurrent PE despite therapeutic anticoagulation or has limited cardiopulmonary reserve that can lead to death from a subsequent PE or when there are contraindications to anticoagulation [61]. Temporary use of IVC filters in patients receiving thrombolytic therapy has been sporadically described; however, more evidence is needed to suggest benefits.

PREPIC2 (the prevention of recurrent pulmonary embolism by vena cava interruption 2) trial compared anticoagulation in addition to IVC filter placement and anticoagulation alone. It included patients presenting with acute PE and DVT with at least 1 criterion for severity (myocardial injury, RVD, DVT, ischemic stroke, active cancer, older than 75 years, chronic cardiorespiratory insufficiency). No difference was found between the 2 groups at 6 months [82]. A sub-analysis of the ICOPER (International Cooperative Pulmonary Embolism Registry) and nationwide inpatient sample analysis of hemodynamically stable PE patients that received thrombolytic therapy and IVC filter had low mortality compared to those who did not [83,84].

A meta-analysis concluded there is a benefit to IVC filter placement in reduction of short-term risk of subsequent PE; however, this increases the risk of DVT in the long run. IVC filters were found to have no impact on overall mortality [85]. IVC filters themselves have complications including filter thrombosis, IVC penetration, perforation, fracture or migration of filter, device tilt, etc. It is, therefore, important to retrieve IVC filters at the earliest when deemed no longer necessary.

### 6.3. Non-Steroidal Anti-Inflammatory Drugs

In the hope to reverse the inflammatory reaction contributing to the RVD in patients with PE, Jimenez et al. assigned intermediate-risk PE patients to receive intravenous diclofenac or IV placebo in addition to standard anticoagulation. This study was stopped prematurely due to slow recruitment. However, an intention to treat analysis showed persistent RVD at 48 h and 7 days [86].

### 6.4. Vasodilators

Pulmonary arterial vasodilators such as inhaled nitric oxide or oral phosphodiesterase inhibitors would lower the pulmonary artery pressure and unload the RV. Kline et al. conducted a randomized placebo-controlled trial where intermediate PE patients were assigned to receive oxygen plus 50 ppm nitrogen (placebo) or oxygen plus 50 ppm nitric oxide (NO) for 24 h. The results were not statistically significant; however, 24% of patients receiving NO reached the primary endpoint of normal RV on echo and plasma troponin less than 14 pg per mL compared to 13% receiving placebo [87].

### 6.5. Newer Treatment Options

Anti-coagulants against factor XI and XII have demonstrated promise in venous thromboembolism prevention and are yet to be studied in the treatment of an established thrombus. Thrombin activatable fibrinolysis inhibitor (TAFI) and alpha2-antiplasmin are newly identified targets that enhance fibrinolysis. Targeted thrombolysis strategies that target specific components of thrombus such as fibrin, coagulation factors, or activated platelets are also in the works. It is still to be established if these novel options will be effective in the context of intermediate-risk PE [88].

## 7. Pulmonary Embolism Response Team (PERT)

PERTs are an initiative that has been suggested for better management of intermediate-risk PE patients. PERT consists of a group of specialists who coordinate the management and complex decision-making for patients with PE. A recent study showed that 91% of the PERT activations were for intermediate-risk PE. We are yet to see if this initiative improves clinical outcomes in PE patients [89].

## 8. Conclusions

The management of patients with intermediate-risk PE is complex due to the absence of extensive evidence regarding interventions and lack of guidelines and calls for a multidisciplinary approach to decision-making. CT/echocardiography, biomarkers, and risk-stratification strategies help in therapeutic decision-making. Patients with intermediate-risk PE should be subcategorized into intermediate-low and intermediate-high risk based on evidence of RVD on imaging or cardiac laboratory biomarker evaluation after the initial assessment and risk stratification. In patients with intermediate-low or intermediate-high risk PE, routine use of primary systemic thrombolysis is not advised. Patients with intermediate-high risk should be closely monitored while on systemic anticoagulation to allow for early detection of hemodynamic decompensation and prompt commencement of rescue reperfusion treatment. If clinical symptoms of hemodynamic decompensation (even without hypotension) emerge during surveillance with systemic anticoagulation, systemic thrombolysis may be considered. If hemodynamic decompensation ensues or is impending in patients with intermediate-high risk PE, catheter directed thrombolysis or systemic thrombolysis with tPA should be considered, and percutaneous mechanical thrombectomy or surgical thrombectomy should be considered if the risk of bleeding under thrombolytic treatment is high.

## Figures and Tables

**Table 1:** Definition of pulmonary embolism based on severity.

	Hemodynamic Instability	RVD or Biomarker Elevation	PESI Class/sPESI Score
**American College of Chest Physicians**			
**Low risk**	No	No	---
**Intermediate risk**	No	Either one present	---
**High risk**	Yes	---	---
**American Heart Association**			
**Low risk**	No	No	---
**Submassive**	No	Either one present	---
**Massive**	Yes	---	---
**European Society of Cardiology**			
**Low risk**	No	No	No
**Intermediate-low risk**	No	Either one positive	PESI Class III-IV or sPESI score ≥ 1
**Intermediate-high risk**	No	Both positive	PESI Class III-IV or sPESI score ≥ 1
**High risk**	Yes	---	PESI Class III-IV or sPESI score ≥ 1

Abbreviations: PESI: pulmonary embolism severity index; RVD: right ventricular dysfunction; sPESI: simplified pulmonary embolism severity index; Legend: Color used to present risk stratification. Green indicates low risk, yellow indicates intermediate-low risk and submassive pulmonary embolism, orange indicates intermediate-high risk and red indicates high risk or massive pulmonary embolism.

**Table 2:** Overview of advantages and disadvantages of major therapeutic strategies utilized in intermediate risk PE.

Treatment Option	Advantages	Disadvantages
**Systemic anticoagulation**	Lower risk of bleeding when rescue therapy is indicated Prevent recurrences with a safety profile that is relatively favorable in terms of major bleeds DOACs have predictable courses and do not require laboratory monitoring Reversible in case of bleeding or a patient requiring procedures	Lack of data on DOACs in intermediate-risk PE patients. Most studies that include DOAC data were stratified on the clot burden, not RV dysfunction or biomarkers
**Systemic thrombolysis**	Rapid administration and pulmonary reperfusion Reduces symptoms and speeds up RV recovery Prevent deterioration in hemodynamic status Lowers mortality	1–5% risk of ICH and the risk may be higher in the elderly population (age > 75)
**Reduced-dose thrombolysis**	Reduced bleeding risk compared to full dose thrombolysis	Increased need for treatment escalation to requiring secondary thrombolysis or CDT
**Catheter-directed therapy**	Reduces symptoms and speeds up RV recovery Lower risk of major bleeding and ICH compared to systemic thrombolysis Some devices include a mechanical embolectomy capability	Limited long-term benefits Could require more time to mobilize
**Surgical embolectomy**	Reduces symptoms and speeds up RV recovery Circumvents requirement for thrombolysis Lower risk of ICH	Limited to the level of segmental pulmonary arteries and more centrally located PE Limited long-term data

Abbreviations: CDT: catheter directed therapy; DOAC: direct oral anticoagulant; ICH: intracranial hemorrhage; PE: pulmonary embolism; RV: right ventricle.

**Table 3:** Overview of major trials comparing the outcomes of thrombolysis with anticoagulation in patients with intermediate-risk pulmonary embolism.

Trials	Groups Compared	Outcomes	Summary
**MAPPET-3, 2002** **[45]**	Heparin with alteplase vs. heparin with placebo	The incidence of the primary outcome (in-hospital death or clinical deterioration requiring an escalation of treatment) was significantly higher in the heparin-plus-placebo group than in the heparin-plus-alteplase group (*p* = 0.006), and the probability of 30-day event-free survival was higher in the heparin-plus-alteplase group (*p* = 0.005) Above variance was due to the higher incidence of treatment escalation in the heparin-plus-placebo group (24.6% vs. 10.2%, *p* = 0.004), since mortality was low in both groups (3.4% in the heparin-plus-alteplase group and 2.2% in the heparin-plus-placebo group, *p* = 0.71). Treatment with heparin plus placebo was associated with almost three times the risk of death or treatment escalation that was associated with heparin plus alteplase (*p* = 0.006) No fatal bleeding or cerebral bleeding occurred in patients receiving heparin plus alteplase	Thrombolytics can prevent clinical decline necessitating the escalation of treatment during the hospital stay
**TIPES, 2010** **[69]**	Tenecteplase group (Tenecteplase with heparin) vs. placebo group (placebo with heparin)	Reduction of RV to LV end-diastolic dimension ratio at 24 h was 0.31 ± 0.08 in patients randomized to tenecteplase as compared to 0.10 ± 0.07 in patients randomized to placebo (*p* = 0.04) One patient randomized to tenecteplase suffered a clinical event (recurrent PE) in comparison to three patients randomized to placebo (1 recurrent PE; 1 clinical decline and 1 non-PE-related mortality) Two nonfatal major bleedings occurred with tenecteplase and one with placebo	Single dose thrombolytics are associated with reduction of RVD at 24 h
**MOPETT, 2013** **[50]**	Lower dose thrombolysis along with anticoagulation vs. anticoagulation alone	Pulmonary hypertension and the composite end point developed in 16% of patients in the thrombolysis group and 57% of patients in the anticoagulation group (*p* < 0.001) and 16% of patients in the thrombolysis group and 63% of patients in the anticoagulation group (*p* < 0.001), respectively The duration of hospitalization was 2.2 ± 0.5 days in the thrombolysis group and 4.9 ± 0.8 days in the anticoagulation group (*p* < 0.001). The combination of death plus recurrent PE was 1.6% in thrombolysis group and 10% in the anticoagulation group (*p* = 0.04) No bleeding occurred in any group, no significant difference was noted in the rate of individual outcomes of death and recurrent PE when assessed independently	Lower dose thrombolysis is safe and effective in the treatment of moderate PE, with a significant immediate reduction in the pulmonary artery pressure that was maintained at 28 months
**PEITHO trial, 2014** **[31]**	Systemic thrombolysis with tenecteplase followed by anticoagulation with heparin vs. heparin alone	Mortality or hemodynamic decompensation occurred in 2.6% of patients in the tenecteplase group as compared with 5.6% in the heparin alone group (*p* = 0.02) Between randomization and day 7, 1.2% in the tenecteplase group and 1.8% in the heparin group died (*p* = 0.42). Extracranial bleeding occurred in 6.3% of patients in the tenecteplase group and 1.2% in the heparin group (*p* < 0.001). Stroke occurred in 2.4% of patients in the tenecteplase group and was hemorrhagic in 10 patients; 0.2% in the heparin group had a stroke, which was hemorrhagic (*p* = 0.003) By day 30, a total of 12 patients (2.4%) in the tenecteplase group and 16 patients (3.2%) in the heparin group had died (*p* = 0.42)	Fibrinolytic therapy prevented hemodynamic decompensation but increased the risk of major hemorrhage and stroke
**TOPCOAT, 2014** **[46]**	Low molecular weight heparin and tenecteplase vs. low molecular weight heparin	The trial was concluded prematurely. Within 5 days, adverse outcomes occurred in 3 placebo-treated patients and 1 tenecteplase-treated patient At 90 days, adverse outcomes occurred in 13 unique placebo-treated patients and five unique tenecteplase-treated patients Thus, 37% of placebo-treated and 15% of tenecteplase-treated patients had at least one adverse outcome (*p* = 0.017)	Treatment with tenecteplase was associated with an increased probability of a favorable composite outcome
**ULTIMA, 2014** **[56]**	Heparin alone vs. heparin with catheter-directed thrombolysis (tPA and USAT)	In the USAT group, the mean RV/LV ratio was reduced from 1.28 ± 0.19 at baseline to 0.99 ± 0.17 at 24 h (*p* < 0.001); in the heparin group, mean RV/LV ratios were 1.20 ± 0.14 and 1.17 ± 0.20, respectively (*p* = 0.31). The mean decrease in RV/LV ratio from baseline to 24 h was 0.30 ± 0.20 versus 0.03 ± 0.16 (*p* < 0.001), respectively At 90 days, there was 1 death (in the heparin group), no major bleeding, 4 minor bleeding episodes (3 in the USAT group and 1 in the heparin group; *p* = 0.61), and no recurrent venous thromboembolism	Standardized USAT regimen was superior to anticoagulation with heparin alone in reversing RV dilatation at 24 h, without an increase in bleeding complications

Abbreviations: LV: left ventricle; PE: pulmonary embolism; RV: right ventricle; RVD: right ventricular dysfunction; tPA: tissue plasminogen activator; USAT: ultrasound-assisted catheter-directed thrombolysis.

## Data Availability

No original data used. All available data is from the public domain.

## Abbreviations

ACCP	American College of Chest Physicians
AHA	American Heart Association
BNP	Brain natriuretic peptide
CDT	Catheter directed therapy
CT	Computed tomography
CTA	Computed tomography angiogram
DVT	Deep vein thrombosis
ECMO	Extracorporeal membrane oxygenation
ESC	European Society of Cardiology
FLARE	FlowTriever Pulmonary Embolectomy clinical study
FLASH	FlowTriever All-Comer Registry for Patient Safety and Hemodynamics
h-FABP	Heart-type fatty acid binding protein
ICOPER	International Cooperative Pulmonary Embolism Registry
IVC	Inferior vena cava
LMWH	Low molecular weight heparin
NO	Nitric oxide
NT-proBNP	N-terminal pro-brain natriuretic peptide
OPTALYSE	Optimal duration of requested pulse thrombolysis procedure and acute intermediate-risk pulmonary embolism
PE	Pulmonary embolism
PEITHO	Pulmonary embolism thrombolysis
PERT	Pulmonary embolism response team
PESI	Pulmonary Embolism Severity Index
PREPIC2	Prevention of recurrent pulmonary embolism by vena cava interruption 2
RCT	Randomized controlled trial
RR	Relative risk
RV	Right ventricular
RVAD	Right ventricular assist device
RVD	Right ventricular dysfunction
SBP	systolic blood pressure
SEATTLE II	EkoSonic endovascular system and Activase for treatment of acute pulmonary embolism
sPESI	Simplified Pulmonary Embolism Severity Index
SUNSET sPE	Standard vs. Ultrasound-Assisted Catheter thrombolysis for Submassive Pulmonary Embolism
TOPCOAT	Tenecteplase or Placebo Cardiopulmonary Outcomes at Three Months
TAFI	Thrombin activatable fibrinolysis inhibitor
tPA	tissue plasminogen activator
ULTIMA	Ultrasound accelerated thrombolysis of pulmonary embolism
USAT	Ultrasound-assisted thrombolysis
